# Bispecific antibody-activated T cells enhance NK cell-mediated antibody-dependent cellular cytotoxicity

**DOI:** 10.1186/s13045-021-01216-w

**Published:** 2021-12-09

**Authors:** Zhaoming Wang, Chaobo Yin, Lawrence G. Lum, Andrean Simons, George J. Weiner

**Affiliations:** 1 Holden Comprehensive Cancer Center, University of Iowa, 5970-Z JPP, 200 Hawkins Drive, Iowa City, IA 52242 USA; 2 Cancer Biology Graduate Program, University of Iowa, Iowa City, IA USA; 3 Department of Internal Medicine, University of Iowa, Iowa City, IA USA; 4 Division of Hematology/Oncology, Department of Medicine, The University of Virginia, Charlottesville, VA USA; 5 Department of Pathology, University of Iowa, Iowa City, IA USA

**Keywords:** NK cell, ADCC, Anti-CD20, Blinatumomab, Bispecific antibody

## Abstract

Resistance to anti-cancer monoclonal antibody (mAb) therapy remains a clinical challenge. Previous work in our laboratory has shown that T cell help in the form of interleukin-2 maintains long-term NK cell viability and NK cell-mediated antibody-dependent cellular cytotoxicity (ADCC). Lack of such T cell help may be a potential mechanism for resistance to mAb therapy. Here, we evaluate whether concomitant treatment with anti-CD3 × anti-cancer bispecific antibodies (bsAbs) can overcome this resistance by enhancing T cell help, and thereby maintaining long-term NK cell-mediated ADCC. Normal donor peripheral blood mononuclear cells were depleted of T cells, replenished with defined numbers of autologous T cells (from 0.75 to 50%) and co-cultured with mono-/bispecific antibody-treated target tumor cells for up to 7 days. At low T cell concentrations, bsAb-activated T cells (mainly CD4^+^ T cells) were more effective than resting T cells at maintaining NK cell viability and ADCC. Brief (4 h to 2 day) bsAb exposure was sufficient to enhance long-term ADCC by NK cells. These findings raise the hypothesis that local T cell activation mediated by systemic treatment with anti-CD3 X anti-cancer bsAb may enhance the anti-tumor efficacy of monospecific mAbs that mediate their primary therapeutic effect via NK-mediated ADCC.

**Supplementary Information:**

The online version contains supplementary material available at 10.1186/s13045-021-01216-w.

## To the editor,

Anti-cancer monoclonal antibodies (mAbs), including rituximab (anti-CD20) and cetuximab (anti-EGFR), are a standard component of cancer therapy. A major mechanism of action of anti-cancer mAbs is NK cell-mediated antibody-dependent cellular cytotoxicity (ADCC) [1, 2]. Resistance to mAb therapy remains a clinical challenge. Our previous work suggests that T cell help, mediated largely by interleukin-2 (IL-2) locally produced by CD4^+^ T cells, maintains long-term NK cell-mediated ADCC and NK number [3]. Thus, lack of adequate T cell help may explain some cases of resistance to mAb therapy.

IL-2 is well known to enhance NK cell activation and ADCC [4, 5]. However, systemic IL-2 administration results in significant toxicity and non-selectively expands regulatory T cells [6, 7], thereby lessening enthusiasm for such combinations. Anti-CD3 x anti-cancer bispecific antibodies (bsAbs) redirect T cell cytotoxicity towards tumor cells [8]. bsAb-activated T cells also produce proinflammatory cytokines, including IL-2 [9, 10]. Here, we explore the hypothesis that bsAb can induce the local production of IL-2 by T cells and maintain NK cell-mediated ADCC.

**Fig. 1:**
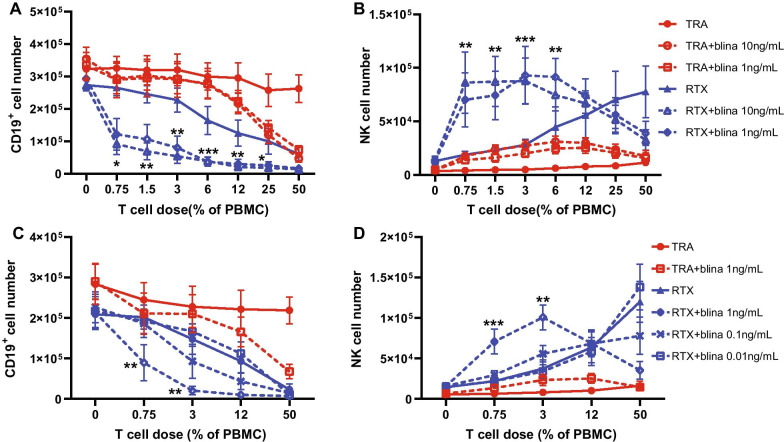
Blinatumomab enhances RTX-mediated NK cell response. PBMCs depleted of CD3^+^ T cells were cocultured with Raji cells and RTX or trastuzumab (TRA) as the control for 7 days. Serial dilutions (from 0.75 to 50% of PBMCs) of autologous CD3^+^ T cells were added back as was blinatumomab to select samples. NK cell response was measured on day 7. **A**, **B** RTX-mediated NK cell elimination of CD19^+^ target cells and NK cell viability increase in a T cell dose-dependent manner. These changes are enhanced by blinatumomab (1 ng/mL or 10 ng/mL) at low T cell percent from 0.75 to 6%. n = 6. **C**, **D** Lower concentrations of blinatumomab at 0.1 ng/mL or 0.01 ng/mL minimally impact on RTX-mediated NK cell ADCC or viability. n = 5. Student’s *t* test was used to calculate statistical significance. *p < 0.05; **p < 0.01; ***p < 0.001 indicate RTX versus RTX + blina 10 ng/mL. *blina* blinatumomab

T cells were depleted from peripheral blood mononuclear cells (PBMCs) and autologous T cells were added back in known concentrations along with target Raji cells, rituximab (RTX) and blinatumomab (anti-CD19 X anti-CD3) and cultured for 1 week (Additional file 2). Blinatumomab at either 1 or 10 ng/mL [11] enhanced elimination of Raji cells by RTX-activated NK cells (Fig. 1A) and increased the number of viable NK cells (Fig. 1B), particularly when lower numbers of T cells were present. By contrast, RTX or blinatumomab alone had minimal impact on NK cells or ADCC when small numbers of T cells were present. The addition of T cells in the trastuzumab control group had little effect on CD19^+^ cell numbers indicating nutrient depletion was not responsible for limiting Raji growth. These results demonstrate that small numbers of T cells activated by blinatumomab enhance RTX-mediated ADCC and NK cell number. The number of viable NK cells was lower in response to RTX plus blinatumomab compared to RTX alone at high T cell concentrations, likely due to the early elimination of target cells and the loss of the RTX-mediated activating signal to NK cells. Concentrations of blinatumomab below 1 ng/ml had limited impact on RTX-mediated NK cell responses (Fig. 1C, D). Similar results were observed with Daudi cells serving as target cells (Additional file 1: Figure S1). Both CD4^+^ and CD8^+^ T cells were able to produce IL-2 in response to blinatumomab (Additional file 1: Figure S2). Blinatumomab-activated CD4^+^ and CD8^+^ T cells enhanced NK cell ADCC and number, with CD4^+^ T cells being more effective at low T cell concentrations (Additional file 1: Figure S3).

**Fig. 2:**
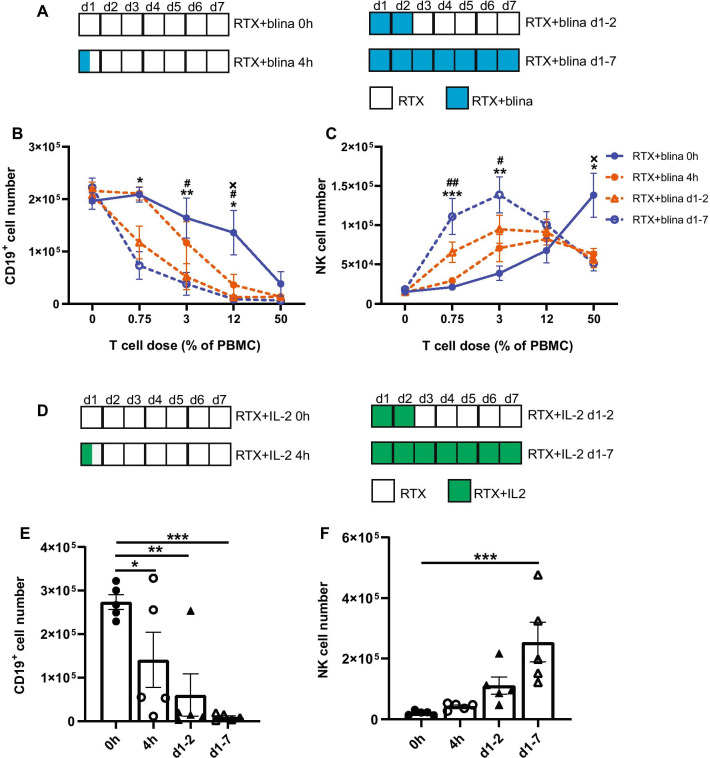
Short-term blinatumomab exposure enhances NK cell ADCC and viability. PBMCs depleted of T cells were cocultured with Raji cells and RTX for 7 days. Serial dilutions (from 0.75 to 50% of PBMCs) of autologous T cells were added back. **A** Blinatumomab (1 ng/mL) was supplemented for the first 4 h (4 h), 2 days (d1-2), 7 days (d1-7) or not added (0 h). After the indicated time, blinatumomab was washed out and the coculture was refreshed with RTX-containing medium. **B**, **C** 2-day blinatumomab enhances RTX-mediated NK cell killing of CD19^+^ target cells and viability. 4-h blinatumomab enhances NK cell ADCC at 12% T cells but fails to increase NK cell viability. n = 6. Student’s *t* test was used to calculate statistical significance. *p < 0.05; **p < 0.01; ***p < 0.001 are the comparisons between RTX + blina d1-7 versus RTX + blina 0 h; #*p* < 0.05, ##*p* < 0.01 indicate RTX + blina d1-2 versus RTX + blina 0 h; × *p* < 0.05 indicates RTX + blina 4 h versus RTX + blina 0 h. **D** PBMCs depleted of T cells were cocultured with Raji cells and RTX for 7 days. Recombinant IL-2 (20 ng/mL) was supplemented for 4 h (4 h), 2 days (d1-2), 7 days (d1-7) or not added (0 h). IL-2 was washed out and replaced by RTX-containing medium after the indicated time. **E**, **F** 2-day or 4-h IL-2 exposure enhances NK cell ADCC of CD19^+^ target cells. Short-term IL-2 treatment also increases the number of viable NK cells, although not statistically significant. n = 5. One-way ANOVA was used to calculate statistical significance. *p < 0.05; **p < 0.01; ***p < 0.001

One major challenge associated with the clinical use of blinatumomab is the need for a continuous 28-day infusion. This led to evaluation of whether short-term blinatumomab exposure can provide enough T help to support RTX-mediated NK cell ADCC. Blinatumomab was added to the culture for various periods of time, then washed out while RTX was maintained for the full 7 days (Fig. 2A). Short-term (4-h or 2-day) blinatumomab exposure enhanced NK cell ADCC and number (Fig. 2B, C). A similar IL-2 washout experiment (Fig. 2D) was performed to explore the role of IL-2 in this process. These results (Fig. 2E, F) are consistent with our prior observation that IL-2 production by T cells is central to providing help for NK-mediated ADCC. Additionally, EGFRBi (anti-EGFR X anti-CD3) [12] enhanced cetuximab-mediated NK cell ADCC, suggesting bsAb-induced T cell help can enhance ADCC mediated by mAb against other targets (Additional file 1: Figure S4).

Collectively, these studies describe a novel mechanism of interaction between mAbs and bsAbs that has the potential to enhance the therapeutic effectiveness of both agents. Anti-CD3 × anti-cancer bsAb induces the local production of cytokines, including IL-2 by T cells which, in turn, enhances NK cell viability and ability to mediate ADCC. It is important to note that PBMCs are an imperfect surrogate for the tumor microenvironment. We considered evaluating this hypothesis in animal models however pilot studies demonstrated significant differences between the human and murine systems. Thus, evaluation of the underlying hypothesis of this manuscript in murine models would be of limited value. A clinical trial is expected to open shortly to further test the hypothesis that intratumoral T cell activation by short-term systemic bsAb treatment could enhance the efficacy of anti-tumor mAb where NK-mediated ADCC is a primary mechanism of action (Additional file 2).

## Supplementary Information

**Additional file 1**. **Supplementary Figures. Figure S1**. Blinatumomab enhances RTX-mediated NK cell response in Daudi cells. **Figure S2.** CD4+ and CD8+ T cells produce IL-2 in response to blinatumomab. **Figure S3.** CD4+ is more efficient than CD8+ T cells in providing help to enhance RTX-mediated NK cell response. **Figure S4.** EGFRBi enhances cetuximab-mediated NK cell response.

**Additional file 2**. Materials and Methods.

## Data Availability

All data are available on reasonable request.

## Abbreviations

ADCC: Antibody-dependent cellular cytotoxicity
bsAb: Bispecific antibody
CTX: Cetuximab
IL-2: Interleukin 2
mAb: Monoclonal antibody
PBMC: Peripheral blood mononuclear cell
RTX: Rituximab
TME: Tumor microenvironment
TRA: Trastuzumab
