# Supplementary material for: Bispecific antibody-activated T cells enhance NK cell-mediated antibody-dependent cellular cytotoxicity
Source: J Hematol Oncol. 2021 Dec 9;14:204. doi: 10.1186/s13045-021-01216-w (PMC8656063; doi:10.1186/s13045-021-01216-w)
Supplement: Supplementary file 1 — Additional file 1. Supplementary Figures. Figure S1. Blinatumomab enhances RTX-mediated NK cell response in Daudi cells. Figure S2. CD4+ and CD8+ T cells produce IL-2 in response to blinatumomab. Figure S3. CD4+ is more efficient than CD8+ T cells in providing help to enhance RTX-mediated NK cell response. Figure S4. EGFRBi enhances cetuximab-mediated NK cell response. [file 13045_2021_1216_MOESM1_ESM.pdf]

**Figure S1**

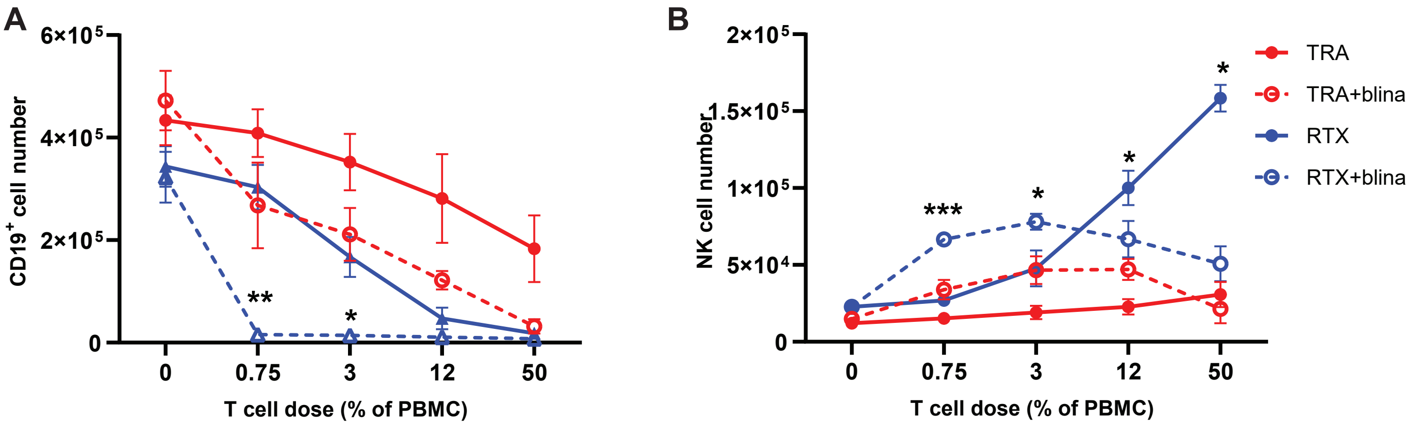


**Figure S1. Blinatumomab enhances RTX-mediated NK cell response in Daudi cells.** PBMCs depleted of T cells were cocultured with Daudi cells and RTX or TRA for 7 days. Serial dilutions (from 0.75% to 50% of PBMCs) of autologous resting or blinatumomab (1ng/mL)-activated T cells were added to the coculture. RTX-mediated NK cell cytotoxicity (**A**) and viability (**B**) are T cell dose-dependent and further enhanced by blinatumomab at low T cell concentrations. Student's t-test was used to calculate statitical significance. n=4. * $p<0.05$, ** $p<0.01$, *** $p<0.001$ indicate RTX+blina versus RTX.

**Figure S2**

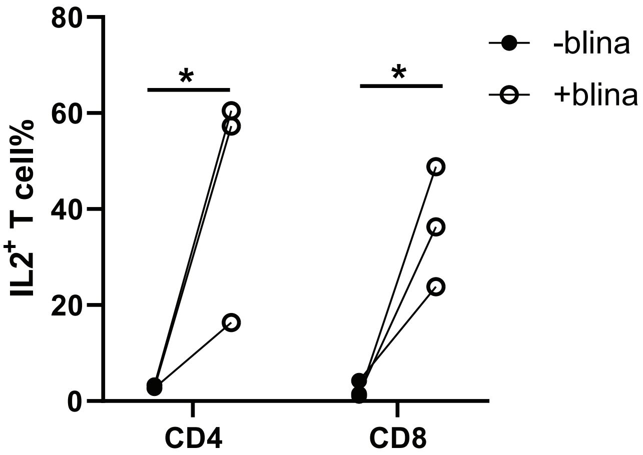


**Figure S2. CD4⁺ and CD8⁺ T cells produce IL-2 in response to blinatumomab.**
PBMCs were cocultured with Raji cells in the presence (+) or absence (-) of blinatumomab (1ng/mL) for 24 hours. Both $CD4^+$ and $CD8^+$ T cells activated by blinatumomab are able to produce IL-2. n=3. * $p<0.05$. blina: blinatumomab.

## Figure S3

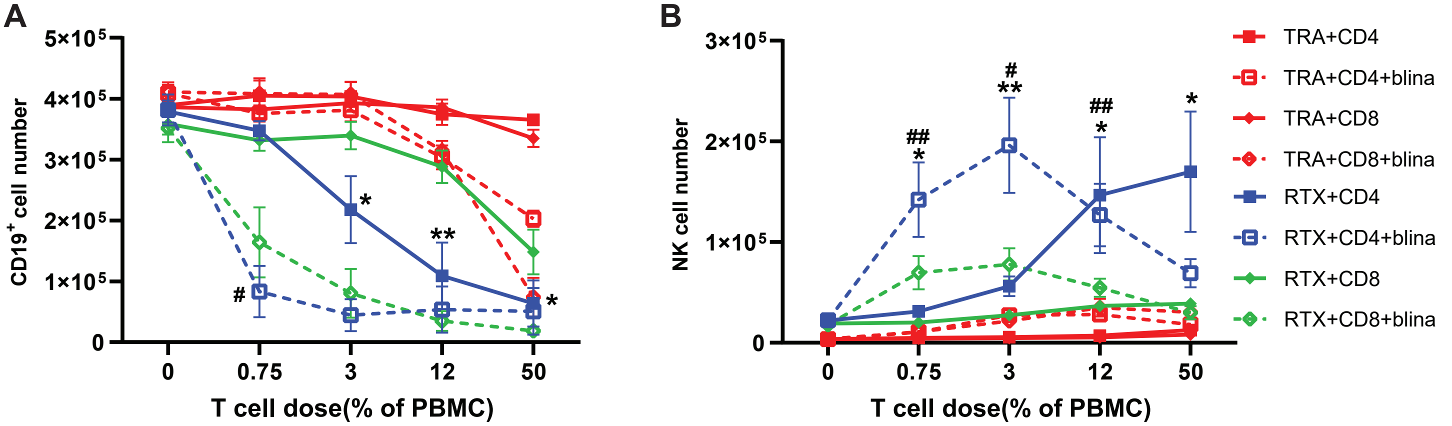


**Figure S3. CD4⁺ is more efficient than CD8⁺ T cells in providing help to enhance RTX-mediated NK cell response.**

PBMCs depleted of T cells were cocultured with Raji cells and RTX or TRA for 7 days. Serial dilutions (from 0.75% to 50% of PBMCs) of autologous $CD4^+$ or $CD8^+$ T cells were added back as was blinatumomab (1ng/mL) to select samples. Resting $CD4^+$ cells were better than $CD8^+$ cells in enhancing elimination of target cells (**A**) and maintaining NK viability (**B**). Both blinatumomab-activated $CD4^+$ and $CD8^+$ T cells were able to enhance RTX-mediated NK ADCC and viability, with CD4 being more efficient at low T cell concentrations. n=5. Student's t-test was used to calculate statitical significance. * $p<0.05$, ** $p<0.01$, *** $p<0.001$ indicate RTX + CD4 versus RTX + CD8; # $p<0.05$, ## $p<0.01$ indicate RTX + CD4 + blina versus RTX + CD8 + blina. blina: blinatumomab.

## Figure S4

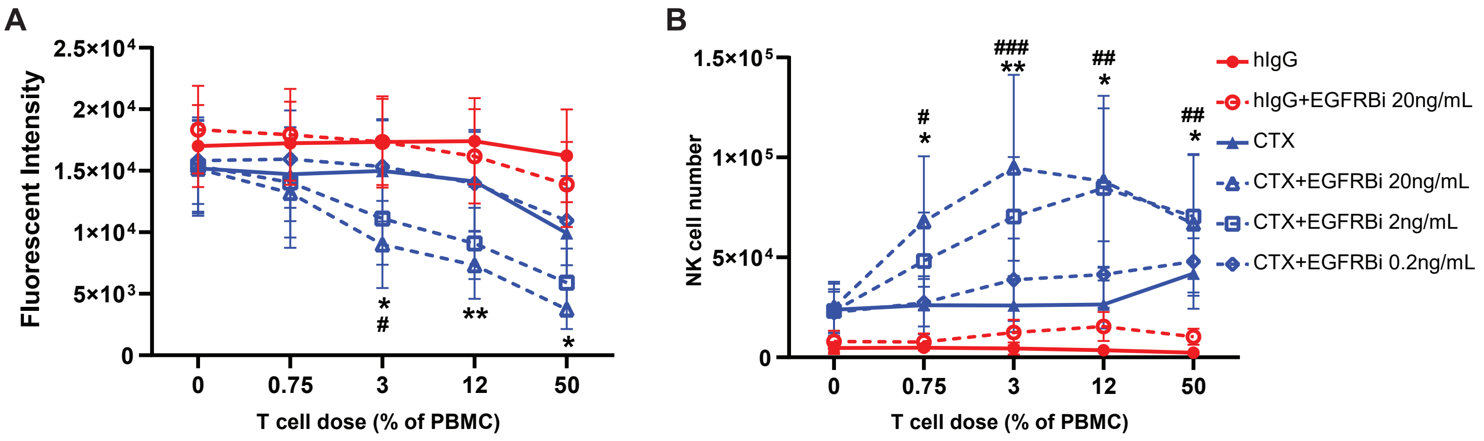


**Figure S4. EGFRBi enhances cetuximab-mediated NK cell response.**
PBMCs depleted of T cells were cocultured with SQ20B cells and cetuximab (CTX) or hIgG for 7 days. Serial dilutions (from 0.75% to 50% of PBMCs) of resting or EGFRBi (20, 2, 0.2ng/mL)-activated autologous T cells were added to the coculture. CTX-mediated NK cell cytotoxicity (**A**) as determined by the resazurin reduction assay and NK viability (**B**) are T cell dose dose-dependent and enhanced by EGFRBi at the concentration of 20ng/mL or 2ng/mL. n=5. Student's t-test was used to calculate statitical significance.
* $p<0.05$, ** $p<0.01$ indicate CTX + bsAb 20ng/mL versus CTX; # $p<0.05$, ## $p<0.01$, ### $p<0.001$ indicate CTX + bsAb 2 ng/mL versus CTX.
