# Supplementary material for: Bispecific antibody-activated T cells enhance NK cell-mediated antibody-dependent cellular cytotoxicity
Source: J Hematol Oncol. 2021 Dec 9;14:204. doi: 10.1186/s13045-021-01216-w (PMC8656063; doi:10.1186/s13045-021-01216-w)
Supplement: Supplementary file 2 — Additional file 2. Materials and Methods. [file 13045_2021_1216_MOESM2_ESM.docx]

**Materials and Methods**

**Samples and reagents.** Peripheral blood mononuclear cells (PBMCs) were isolated from leukocyte reduction system cones from healthy donors (the DeGowin Blood Center, the University of Iowa) using Ficoll-based density centrifugation. B-cell lymphoma cell lines – Raji and Daudi cells were purchased from the American Type Culture Collection. The squamous cell carcinoma cell line - SQ20B was provided by Dr. Andrean Simons-Burnett at the University of Iowa. Rituximab (RTX), trastuzumab (TRA), cetuximab (CTX) were from University of Iowa Hospitals & Clinics. Anti-CD3 x anti-EGFR bsAb (EGFRBi) was produced by Dr. Lawrence Lum^1^ as previously described. Blinatumomab was obtained from Amgen (Thousand Oaks, California). Control human IgG_1_ kappa was from Sigma. Recombinant human IL-2 was from Peprotech. RPMI supplemented with 10% fetal bovine serum, 2 mM L-Glutamine, 50 µM 2-mercaptoethanol, 100 U/mL penicillin and 100 µg/ml streptomycin was used to culture PBMC, Raji and Daudi cells. DMEM supplemented with 10% fetal bovine serum, 100 U/mL penicillin and 100 µg/mL streptomycin was used to culture SQ20B cells.

**T cell depletion and isolation.** Cell depletion and isolation kits were from Miltenyi Biotec. CD3^+^ T cells were depleted from PBMCs using CD3 microbeads. Untouched CD3^+^ / CD4^+^ / CD8^+^ T cells were isolated from PBMCs using Pan T cell / CD4^+^ T cell / CD8^+^ T cell isolation kits per the manufacturer’s instructions.

**Coculture assays.** PBMCs were depleted of T cells, and 10^6^ non-T cells were added as effector cells to target cells. Target cells consisted of 0.2 million to 0.4 million Raji, Daudi (B-cell lymphoma cell line) or SQ20B (head and neck cancer cell line). Then defined numbers of autologous T cells (from 0.75% to 50% of PBMCs) were added back to the coculture in a total volume of 200 µL in U bottom 96-well plates. The target-to effector ratio of non-T cells is constant in this setting while the number of T cells varies. MAb, bsAb or cytokine was then added including RTX and TRA at 1ug/mL, CTX and hIgG at 2ug/mL, IL-2 at 20ng/mL and bsAb including blinatumomab or EGFRBi at the indicated dose.

**Flow cytometry analysis**. The staining antibodies (BioLegend) included anti-human CD3-APC/Cy7 (HIT3a), CD56-A647 (HCD56), CD14-A700 (HCD14), CD19-PerCP (HIB19), CD16-Pacific Blue (3G8), CD25-FITC (M-A251), IL2 (MQ1-17H12). Immunostaining was performed as described^2^. Briefly, cells were first washed three times with PBS and stained with Zombie Aqua at room temperature for 15 minutes to distinguish live from dead cells. Cells were then incubated with staining antibodies at 4ºC for 15 minutes and fixed in 2% paraformaldehyde. For intracellular staining, brefeldin A was added for the last 6 hours of co-culture. After surface staining, cells were fixed and permeabilized using the Cyto-Fast Fix / Per Buffer Set (BioLegend), followed by staining in the permeabilization buffer at room temperature for 30 minutes. Samples were read by the Cytek Aurora flow cytometer (Cytek Biosciences) within 24 hours. Data were analyzed using the FlowJo software (FlowJo LLC.).

**ADCC quantification.** For studies with anti-CD20, ADCC was determined by analyzing the number of remaining viable Raji and Daudi cells using flow cytometry and measuring remaining CD19^+^ target cells. For adherent SQ20B cells, 96-well plates were washed with PBS three times to remove cells in suspension. A resazurin reduction assay was used to quantitatively measure the viability of remaining adherent tumor cells as described^3^. Briefly, 200 µL resazurin (StemCell technologies) reconstituted at the concentration of 44 µM was added to each well and incubated at 37ºC for 2 hours. The plate was read by spectrometer at excitation at 530 nm and emission at 590 nm.

**Statistical analysis.** Data was presented as mean ± SEM. Two-tailed Student’s t-test was used to compare two independent groups. One-way ANOVA was used to compare multiple groups. All analyses were performed using GraphPad Prism8 (GraphPad Software Inc.). p<0.05 was considered to be statistically significant.

**Reference**

1 Lum, L. G. *et al.* Clinical and immune responses to anti-CD3 x anti-EGFR bispecific antibody armed activated T cells (EGFR BATs) in pancreatic cancer patients. *Oncoimmunology* **9**, 1773201, doi:10.1080/2162402X.2020.1773201 (2020).

2 Wang, Z., Chimenti, M. S., Strouse, C. & Weiner, G. J. T cells, particularly activated CD4(+) cells, maintain anti-CD20-mediated NK cell viability and antibody dependent cellular cytotoxicity. *Cancer Immunol Immunother*, doi:10.1007/s00262-021-02976-7 (2021).

3 Uzarski, J. S., DiVito, M. D., Wertheim, J. A. & Miller, W. M. Essential design considerations for the resazurin reduction assay to noninvasively quantify cell expansion within perfused extracellular matrix scaffolds. *Biomaterials* **129**, 163-175, doi:10.1016/j.biomaterials.2017.02.015 (2017).
